# Sodium-Glucose Cotransporter-2 Inhibitors Across the Glycemic Spectrum: Cardiovascular and Renal Outcomes With Mechanistic Insights

**DOI:** 10.7759/cureus.105114

**Published:** 2026-03-12

**Authors:** Raghad Abdelfadil Elgazzar, Mahmoud Ramadan

**Affiliations:** 1 College of Medicine, University of Sharjah, Sharjah, ARE; 2 Cardiology, University of Sharjah, Sharjah, ARE

**Keywords:** canagliflozin, cardiovascular outcomes, chronic kidney disease, dapagliflozin, empagliflozin, heart failure, natriuresis, renal outcomes, sglt2 inhibitors, type 2 diabetes mellitus

## Abstract

Sodium-glucose cotransporter 2 (SGLT2) inhibitors were initially developed as glucose-lowering agents for type 2 diabetes mellitus (T2DM) by reducing proximal tubular glucose reabsorption and promoting glucosuria. Subsequent cardiovascular and renal outcome trials established that these agents provide clinically meaningful benefits that extend beyond glycemic control, including reductions in heart failure (HF) events and slowing of chronic kidney disease (CKD) progression in patients with and without diabetes. We conducted a narrative literature review using PubMed and Google Scholar to identify English-language studies published between January 2019 and December 2025 evaluating SGLT2 inhibitors (empagliflozin, dapagliflozin, canagliflozin, ertugliflozin) across cardiovascular and renal outcomes. Eligible evidence included randomized controlled trials, large observational studies, systematic reviews/meta-analyses, guidelines, post‑hoc exploratory analyses, secondary analyses, and mechanistic/preclinical studies. Mechanistic data support benefits through natriuresis and osmotic diuresis with favorable ventricular loading, restoration of tubuloglomerular feedback and reduced intraglomerular pressure, improved myocardial energetics, and attenuation of oxidative stress and inflammation. Clinically, SGLT2 inhibitors consistently reduce HF hospitalizations and composite cardiorenal endpoints in diabetic and non-diabetic populations across CKD stages studied and heart conditions, with a generally favorable safety profile; genital mycotic infections are most common, while diabetic ketoacidosis and volume depletion are uncommon with appropriate patient selection and counselling. Evidence gaps remain for stage 5 CKD and dialysis populations and for defining outcomes in lower-risk post-myocardial infarction cohorts. Overall, current data support SGLT2 inhibitors as foundational therapy for eligible patients with HF and/or CKD irrespective of diabetes status.

## Introduction and background

The first sodium-glucose transporter (SGLT) inhibitor drug, phlorizin, was isolated from apple tree bark in 1836. Its capacity to induce diuresis and glucosuria, demonstrated in 1885, prompted early investigations of its renal and metabolic effects. Two SGLT isoforms were identified late in the 20th century via phlorizin's in vitro studies. It inhibits glucose reabsorption in the intestine and renal proximal tubule: SGLT1 (intestinal) and SGLT2 (renal). The first orally active phlorizin-derived SGLT inhibitors were developed because of observations that phlorizin controlled glucose levels and decreased insulin resistance in diabetic animal models [1]. SGLT2 inhibitors are one of the subtypes of glucose-lowering medications that reduce glucose reabsorption from the renal filtrate, hence eliminating excess glucose by a glucosuric action [2]. The sodium-dependent glucose transporter-2 (SGLT2), which is found in the early proximal tubule of the kidney, is the primary mechanism for the reabsorption of glucose filtered by the kidney glomeruli. As a result, glucose is leaked into the urine by SGLT2 inhibitors. This provided the primary basis for the development of these compounds as drugs that reduce blood glucose levels in individuals with type 2 diabetes mellitus (T2DM) [3]. SGLT2 inhibitors were first associated with concerns about possible adverse effects on the renal system, particularly worsening acute kidney injury, increasing genitourinary infections, and impairing bladder function [2]. However, it has been demonstrated that SGLT2 inhibitors have another action beyond the glucose-lowering effect, such as significantly improving kidney and cardiovascular (CV) health. Multiple meta-analyses and systematic reviews concluded that SGLT2 inhibitors significantly improve CV health. Overall, these analyses demonstrate that SGLT2 inhibitors reduce major CV events, improve outcomes in heart failure (HF) populations (including reducing mortality, hospitalizations, and composite CV events), and improve the CV and renal health of high-risk diabetic patients, with safety risks usually being outweighed by clinical benefits [4-7]. Both European and American guidelines prescribe SGLT2 inhibitors because they lower the risk of the combined endpoint of CV death and hospitalizations in patients with HF, regardless of ejection fraction [8-10]. SGLT2 inhibitors significantly reduce renal disease progression and mortality in patients with diabetic kidney disease. Regarding chronic kidney disease (CKD), SGLT2 inhibitors provide strong CV and renal protection down to guideline-specified estimated glomerular filtration rate (eGFR) thresholds [11,12]. These results support guideline recommendations to prioritize SGLT2 inhibitors in this high‑risk population [13,14]. The aim of this review is to synthesize the most recent data about the effects and mechanisms of SGLT2 inhibitors on CV and renal outcomes in various glycemic states.

## Review

Methodology

A structured literature search was conducted in PubMed and Google Scholar for articles published between January 2019 and December 2025, focusing on recent SGLT2 inhibitor evidence in CKD and CV outcomes. The search was completed in December 2025. The search strategy combined the following keywords using Boolean operators: (“SGLT2 inhibitor” OR “empagliflozin” OR “dapagliflozin” OR “canagliflozin” OR “ertugliflozin”) AND (“chronic kidney disease” OR “cardiovascular outcomes” OR “heart failure” OR “type 2 diabetes”). Filters were applied to include articles published in English.

Eligible studies included randomized controlled trials, large observational studies, clinical guidelines, systematic reviews, meta-analyses, post‑hoc exploratory analyses, secondary analyses, and relevant preclinical (animal or mechanistic) studies evaluating CV and renal outcomes. Case reports, small case series, editorials, commentaries, conference abstracts without full text, and studies not reporting relevant outcomes were excluded.

From 88 articles initially screened by title/abstract, 23 were excluded after full-text review, yielding 65 studies for this narrative review. A formal Preferred Reporting Items for Systematic Reviews and Meta-Analyses (PRISMA)-guided systematic protocol was not followed.

Effects of SGLT2 inhibitors on CV outcomes

SGLT2 inhibitors improve CV outcomes in individuals with and without diabetes across conditions such as HF, myocardial infarction (MI), and endothelial dysfunction [15]. In 2008, the US Food and Drug Administration (FDA) required that new antidiabetic therapies be evaluated not only for glycemic control but also for their CV outcomes [16].

Hemodynamic Effects and Ventricular Loading Conditions

Preload reduction, neurohormonal damping, and renal perfusion maintenance work together to improve systolic and diastolic performance across the subtypes of HF by shifting the ventricles into a lower-stress loading state [17]. SGLT2 inhibitors reduce cardiac preload and afterload by osmotic diuresis and natriuresis, resulting in lower plasma volume and total body sodium, without significantly activating the renin-angiotensin system or the sympathetic nervous system, which reduces ventricular wall stress [16]. According to a preclinical study, the mechanism by which the SGLT2 inhibitor induces natriuresis is mostly mediated via the proximal tubular Na+/H+ exchanger NHE3. They observed that low-dose empagliflozin acutely increases urinary sodium, flow, and bicarbonate excretion independent of glucosuria. They also noticed the effects were absent in NHE3-deficient models. Chronic, empagliflozin lowers systolic blood pressure in an NHE3-dependent manner and increases inhibitory phosphorylation of NHE3 (S552/S605), which will cause an increase in sodium excretion, particularly in diabetic states. In conclusion, combining all these data shows that SGLT2 inhibition reduces cardiac preload and afterload by enhancing sodium and water excretion via NHE3 effects and osmotic diuresis [18].

Metabolic Shifts and Myocardial Energetics

In stressful conditions like HF and T2DM, impaired myocardial glucose utilization forces the heart to depend predominantly on fatty acid oxidation, which is an inefficient energy process that causes an increased oxygen demand. This metabolic change impairs cardiac relaxation and contributes to the development of diastolic dysfunction because it increases oxidative stress and interferes with cardiomyocytes' ability to handle calcium [19]. Ketone bodies provide an efficient alternative energy source for the heart. Research has demonstrated that β-hydroxybutyrate can increase cardiac output and diastolic performance, which helps reverse the action of ventricular remodelling, and generate anti-inflammatory effects [20,21]. SGLT2 inhibitors induce metabolic changes that increase circulating ketone body availability. That happens by several mechanisms. First, it reduces renal glucose reabsorption. These drugs lower plasma glucose and insulin levels, which promotes lipolysis and ketone production. Secondly, increased glucagon further enhances ketogenesis, while reduced renal excretion of ketones increases their systemic availability. The resulting rise in ketone bodies provides a more efficient energy source for the failing heart, improving cardiac energy efficiency and potentially slowing HF progression [16]. This mechanism is supported by experimental evidence from a preclinical study from a non-diabetic pig model of HF, which demonstrates better systolic function and ventricular remodelling with empagliflozin administration [22]. However, it remains unclear whether these ketone-related changes represent true intermediate causal mediators of clinical benefit or merely occur alongside them as an adaptive, epiphenomenal response of the failing myocardium.

This shift enhances myocardial adenosine triphosphate (ATP) production while reducing oxygen consumption, thereby improving cardiac efficiency in energy-starved failing hearts [23]. In patients with HF who do not have diabetes, improved myocardial energetics helps to reduce adverse ventricular remodelling and improve contractile performance [16].

Mitochondrial Function and Oxidative Stress Modulation

Mitochondria detect environmental stress and help cells adapt by regulating energy production and reactive oxygen species (ROS) generation, which in turn influences endothelial cell growth, proliferation, and senescence [24]. At the molecular level, SGLT2 inhibitors improve mitochondrial function by enhancing mitochondrial biogenesis and preserving mitochondrial membrane integrity in cardiomyocytes [25]. Preclinical study shows that dapagliflozin and empagliflozin improve cardiac mitochondrial function and reduce oxidative stress through complementary mechanisms, involving activation of SIRT1/PGC-1α [26] and inhibition of TGF-β/Smad with activation of Nrf2/ARE [27], respectively. These agents reduce mitochondrial ROS production, thereby limiting oxidative damage to proteins, lipids, and mitochondrial DNA within the myocardium [25]. The reduction in oxidative stress improves endothelial and cardiomyocyte signalling and restores myocardial cellular homeostasis, which is particularly relevant in HF pathophysiology [15].

Endothelial Function and Microvascular Integrity

Nitric oxide synthase (NOS) produces nitric oxide, a key vasodilatory and anti-atherosclerotic molecule essential for endothelial health [28]. Decrease in availability of endothelial-dependent nitric oxide promotes fibroblast proliferation, extracellular matrix deposition, and impairs cardiomyocyte relaxation. Thus, improved endothelial nitric oxide bioavailability enhances coronary microvascular perfusion, which is critical for myocardial oxygen delivery in HF [29]. Through several coordinated molecular mechanisms, SGLT2-inhibitor therapy enhances endothelium health [30]. Preclinical study demonstrates that dapagliflozin and canagliflozin increase AMPK phosphorylation in human coronary microvascular and umbilical vein endothelial cells, which restricts endothelial apoptosis, controls oxidative stress, and lowers pro-inflammatory signalling [31,32]. Overall, this results in an anti-thrombotic endothelium phenotype that promotes organ perfusion and slows the development of nephropathy and cardiomyopathy. SGLT2 inhibition (empagliflozin) diminishes glucose-driven ROS generation, prevents angiotensin‑II‑mediated excessive oxidative stress, and maintains nitric‑oxide bioavailability, which together protect endothelial cells from injury and functional decline [33].

Reduction of Autosis and Ischemic Myocardial Injury

Although mortality from acute MI has declined over the past 25 years, the risk of developing HF after MI and long-term mortality, extending beyond one year, remains significant [34]. Clinical needs for cardioprotection in addition to early reperfusion to decrease the size of the infarct and the incidence and extent of HF after MI are still not met [35]. At the molecular level, coronary microvascular dysfunction is primarily driven by oxidative stress. The pro-apoptotic adaptor p66Shc is activated when NADPH oxidase and mitochondria overproduce ROS, which in turn increases the production of ROS.

Elevated ROS impairs nitric oxide-mediated vasodilation and enhances endothelin-1-induced vasoconstriction through the RhoA/Rho-kinase pathway [29,36]. Preclinical study demonstrates that empagliflozin protects the heart from MI by reducing cardiomyocytes from autophagic cell death. Empagliflozin improved cardiac function and survival in mouse models by reducing infarct size and fibrosis. At the molecular level, this occurs as empagliflozin inhibits Na^+^/H^+^ exchanger-1 in cardiomyocytes, preventing excessive autophagy and revealing a glucose-independent cardioprotective pathway. However, the NHE1-autosis pathway is supported primarily by preclinical experimental models, and its translational relevance to the clinical benefits of SGLT2 inhibitors in human HF remains uncertain.

NHE1 is a key regulator of intracellular pH and ionic balance, and its overactivation promotes excessive autophagy leading to autosis [37]. Empagliflozin has been shown to reduce myocardial injury even in the absence of SGLT2 in cardiac tissue, suggesting that its main mechanism of action as a cardioprotective depends on these additional pathways [38]. Inhibiting autosis reduces post-infarction ventricular dysfunction and maintains cardiomyocyte viability during acute ischemic episodes [38,34].

Effects of SGLT2 inhibitors in CKD

SGLT2 is a low-affinity, high-capacity glucose transporter located on the luminal membrane of the S1 and S2 segments of the proximal tubule, where it is responsible for reabsorbing approximately 97% of filtered glucose. In contrast, SGLT1 is a high-affinity, low-capacity transporter situated in the S3 segment of the proximal tubule and accounts for the reabsorption of the remaining glucose. Beyond the kidney, SGLT1 is also expressed in the jejunum, the thick ascending limb of the loop of Henle, the macula densa, and myocardial capillaries. In diabetes mellitus, the expression of both SGLT transporters is upregulated.

Blocking SGLT2 in the S1-S2 proximal tubule, which normally reabsorbs filtered glucose, with SGLT1 in the S3 segment handling the remainder, prevents reabsorption of this glucose load during inhibition. Increased glucose and sodium excretion ensue as downstream SGLT1 partially compensates, creating osmotic diuresis that lowers plasma volume and total-body sodium [39]. SGLT2 inhibitors induce natriuresis via dual inhibition of sodium-glucose cotransport and NHE3 [40]. NHE3 is a major regulator of sodium reabsorption and is upregulated by RAAS activation in HF and T2DM [41]. The natriuretic effect is mediated in part by direct inhibition of the sodium‑hydrogen exchanger‑3, which shares structural overlap with SGLT2 on the apical surface of renal epithelial cells [18]. However, NHE3 inhibition, primarily supported by preclinical data, may contribute to natriuresis in an experimental context.

Unlike loop diuretics, the natriuretic and diuretic effects of SGLT2 inhibitors are transient and typically do not trigger marked neurohormonal activation, electrolyte disturbances, or kidney dysfunction, though mild responses may occur. The mechanism potentially involving a proposed plasma volume set-point reset remains under investigation. SGLT2 inhibitors mainly promote diuresis in hypervolemic states, while preserving sodium and water when volume is normal. This is due to their proximal tubular site of action, which increases salt delivery to the macula densa and suppresses RAAS, allowing reduction of intraglomerular pressure to reabsorb sodium appropriately when needed [17]. Altered tubuloglomerular feedback leading to glomerular hyperfiltration is a key mechanism driving CKD progression, particularly in diabetes, and similar hemodynamic disturbances also occur in non-diabetic CKD. By increasing distal sodium delivery to the macula densa, SGLT2 inhibitors restore tubuloglomerular feedback, reduce intraglomerular pressure, and normalize single-nephron hyperfiltration. This results in an initial, reversible decline in GFR like that seen with renin-angiotensin system inhibitors, which reflects beneficial hemodynamic effects rather than kidney injury [42].

Preclinical studies show that SGLT2 inhibition reduces the production of pro-inflammatory and pro-fibrotic mediators, such as nuclear factor-κB (NFκB), tumor necrosis factor receptor 1 (TNFR1), interleukin-6 (IL-6), matrix metalloproteinase-7 (MMP-7), and fibronectin-1 [43]. The net effect is attenuation of renal scarring and slowing of CKD progression. Luminal glucose competes with uric acid for reabsorption via the proximal tubular urate transporter-1 (URAT-1). By increasing intraluminal glucose concentrations, SGLT2 inhibitors reduce uric acid reabsorption and may also directly inhibit URAT-1, leading to a substantial increase in renal uric acid excretion. Because uric acid promotes oxidative stress, inflammation, vascular smooth muscle proliferation, and renal injury, the urate-lowering effect of SGLT2 inhibitors may help preserve vascular and renal structure and function [39].

Clinical evidence of SGLT2 inhibitors’ CV benefits

Randomized controlled trials have shown that SGLT-2 inhibitors provide CV benefits across a variety of patient populations (Table 1), including acute MI, chronic HF, and high-risk T2DM and non-diabetic populations as well [44-46]. The EMPA-REG and CANVAS Programs trials showed significant CV risk reduction with SGLT2 inhibition in individuals with T2DM and existing CV disease, establishing HF as a major therapeutic target [44,45]. The EMPA-REG OUTCOME trial enrolled approximately 7,020 participants randomized to empagliflozin (10 mg or 25 mg) or placebo in addition to standard care. Empagliflozin significantly reduced the primary composite endpoint of three-point major adverse cardiovascular events (MACE) (HR 0.86; 95% CI 0.74-0.99).

**Table 1:** Key clinical trials of SGLT2 inhibitors on cardiovascular outcomes across the glycemic spectrum SGLT2: sodium-glucose cotransporter 2; CV: cardiovascular; CVD: cardiovascular disease; FU: follow-up; HF: heart failure; HFH: heart failure hospitalization; HFrEF: heart failure with reduced ejection fraction; HFmrEF: heart failure with mildly reduced ejection fraction; HFpEF: heart failure with preserved ejection fraction; HR: hazard ratio; LVEF: left ventricular ejection fraction; MACE: major adverse cardiovascular events; MI: myocardial infarction; NT-proBNP: N-terminal pro-B-type natriuretic peptide; T2DM: type 2 diabetes mellitus

Trial	n	T2DM %	Median FU (years)	Key findings	Main results
EMPA-REG OUTCOME/secondary analysis [44]	7,020	100	3.1	Reduced CV death and HF hospitalization in T2DM with CVD	MACE: HR 0.86 (0.74-0.99) HFH: HR 0.65 (0.50-0.85) CV death: HR 0.62 (0.49-0.77)
CANVAS/secondary analysis [45]	10,000	100	2.0	Reduced HF hospitalization in T2DM	HFH: HR 0.67 (0.52-0.87)
DELIVER [46]	6,263	50	2.3	Reduced worsening HF in HFmrEF/HFpEF	Worsening HF or CV death: HR 0.82 (0.73-0.92)
EMPULSE [47]	530	47	0.25	Improved clinical status in acute HF	Win ratio 1.36 (1.09-1.68) CV death/HF event: HR 0.69 (0.45-1.08)
EMMY [48]	476	13	0.5	Early empagliflozin after MI improved cardiac remodelling markers	NT-proBNP reduction by 15% vs placebo (p=0.026) LVEF increase by 1.5% (95% CI 0.2-2.9)
DAPA-MI [49]	4,017	0	0.97	No reduction in hard CV outcomes post-MI	CV death/HFH: HR 0.95 (0.64-1.40) New T2DM: HR 0.53 (0.36-0.77)
DAPA-HF [50]	4,744	42	≈1.5	Reduced CV death/HFH in HFrEF	HR 0.74 (0.65-0.85) (CV death/HFH)
EMPEROR-Reduced [51]	3,730	49	1.3	Reduced CV death/HFH in HFrEF	HR 0.75 (0.65-0.86) (CV death/HFH)
EMPEROR-Preserved [52]	5,988	49	2.2	Reduced HFH in HFpEF	HR 0.79 (0.69-0.90) (HFH)
DECLARE-TIMI 58 [53]	17,160	100	4.2	Reduced HFH in T2DM with CVD	HR 0.73 (0.61-0.88) (HFH)

The most pronounced effects were observed in CV mortality and HF outcomes. CV death was reduced by 38% (HR 0.62; 95% CI 0.49-0.77), HF hospitalization by 35% (HR 0.65; 95% CI 0.50-0.85), and all-cause mortality by 32% (HR 0.68; 95% CI 0.57-0.82). Mediation analyses suggested that nearly half of the reduction in HF hospitalization and HF-related death was independent of glycemic control, blood pressure, or lipid changes, supporting mechanisms related to volume contraction, natriuresis, and renal protection rather than glucose lowering alone [44]. Similarly, the CANVAS Program integrated data from approximately 10,000 participants randomized to canagliflozin or placebo, with a mean follow-up of about two years. Canagliflozin significantly reduced hospitalization for HF (HR 0.67; 95% CI 0.52-0.87).

Mechanistic analyses indicated that the HF benefit was largely mediated by increases in erythrocyte count and hemoglobin, reductions in serum urate, and improvements in albuminuria, suggesting combined volume-related, hematopoietic, and uric acid-lowering effects [45]. In both trials, SGLT2 inhibition was associated with modest reductions in glycated hemoglobin (HbA1c), body weight, and systolic blood pressure. Lipid changes were generally small, including modest increases in LDL-cholesterol and minor alterations in HDL-cholesterol and triglycerides. Additionally, serum urate levels were reduced, particularly with canagliflozin [44,45].

In chronic HF populations, the DELIVER trial evaluated dapagliflozin in 6,263 patients with HF with mildly reduced or preserved ejection fraction (HFmrEF/HFpEF), randomized to dapagliflozin or placebo. Over a median follow-up of approximately 2.3 years, dapagliflozin significantly reduced the primary composite endpoint of worsening HF or CV death (HR 0.82; 95% CI 0.73-0.92; P<0.001). The reduction was primarily driven by a decrease in worsening HF events (HR 0.79; 95% CI 0.69-0.91), while CV death showed a non-significant trend toward reduction (HR 0.88; 95% CI 0.74-1.05). Importantly, the benefit was consistent across subgroups, including patients with left ventricular ejection fraction (LVEF) ≥60% and <60%, and irrespective of diabetes status [46].

In the acute setting, the EMPULSE trial investigated the initiation of empagliflozin in 530 patients hospitalized for acute HF, regardless of ejection fraction or diabetes status. Over 90 days, empagliflozin significantly improved a hierarchical composite outcome incorporating all-cause death, HF events, and change in symptom burden (win ratio 1.36; 95% CI 1.09-1.68; P<0.01). Although the reduction in CV death or HF events did not reach statistical significance (HR 0.69; 95% CI 0.45-1.08), patients experienced clinically meaningful improvements in health status, reflected by an increase of 4.45 points in the Kansas City Cardiomyopathy Questionnaire total symptom score (KCCQ-TSS). Additionally, N-terminal pro B-type natriuretic peptide (NT-proBNP) levels were significantly reduced (geometric mean ratio 0.90; 95% CI 0.82-0.98) [47].

The cardioprotective effects of SGLT2 inhibition have also been explored in the post-acute MI setting in the EMMY trial. This study randomized approximately 476 patients with large MI who underwent percutaneous coronary intervention (PCI) to empagliflozin or placebo within 72 hours of revascularization. At six months, empagliflozin led to a 15% greater reduction in NT-proBNP compared with placebo (P=0.026). Modest but significant improvements in cardiac structure and function were observed, including an increase in LVEF (+1.5%; 95% CI 0.2-2.9%; P=0.029), reduction in the E/e′ ratio (-6.8%; 95% CI 1.3-11.3%; P=0.015), and decreases in left ventricular end-systolic and end-diastolic volumes (LVESV -7.5 mL; LVEDV -9.7 mL) [48].

The DAPA-MI trial evaluated dapagliflozin in 4,017 patients with recent MI but without prior diabetes or HF. Over a median follow-up of approximately one year, dapagliflozin improved the hierarchical composite primary outcome (win ratio 1.34; 95% CI 1.20-1.50; P<0.001), reflecting overall cardiometabolic benefit. However, dapagliflozin did not significantly reduce hard CV endpoints. The risk of CV death or HF hospitalization was neutral (HR 0.95; 95% CI 0.64-1.40), with no significant differences observed in broader composite outcomes including CV death, HF, or recurrent MI (HR 0.95; 95% CI 0.70-1.29), MACE (HR 0.94; 95% CI 0.67-1.31), or all-cause mortality (HR 1.22; 95% CI 0.77-1.92). Notably, dapagliflozin significantly reduced the incidence of new-onset T2DM (HR 0.53; 95% CI 0.36-0.77) and promoted modest weight reduction (-1.65 kg vs. +0.24 kg with placebo). Overall, DAPA-MI suggests that in patients without established diabetes or HF, early post-MI initiation of dapagliflozin confers favorable cardiometabolic effects but does not significantly reduce short-term major CV outcomes [49].

The DAPA-HF trial enrolled 4,744 patients with HFrEF (LVEF ≤40%), of whom 42% had T2DM at baseline. Over a median follow-up of 18.2 months (~1.5 years), dapagliflozin reduced the primary composite of CV death or worsening HF (hospitalization/urgent visit) by 26% (HR 0.74, 95% CI 0.65-0.85; p<0.001).

Secondary outcomes showed consistent benefits, including lower hospitalization for HF or CV death (HR 0.75, 95% CI 0.65-0.85). Dapagliflozin also improved KCCQ symptom scores more than placebo. This landmark trial established the efficacy of SGLT2 inhibitors in non-diabetic HFrEF, expanding their therapeutic role beyond glucose lowering [50].

EMPEROR-Reduced (n=3,730, 49% T2DM, 1.3y follow-up) replicated these findings with empagliflozin in HFrEF (LVEF ≤40%), yielding 25% reduction in CV death or HF hospitalization (HR 0.75, 95% CI 0.65-0.86, p<0.001). HF hospitalizations drove the benefit (HR 0.69), with consistent effects across diabetes subgroups and established therapies (angiotensin-converting enzyme inhibitors (ACEi) or angiotensin receptor blockers (ARB), beta-blockers, and mineralocorticoid receptor antagonists) [51].

The EMPEROR-Preserved trial enrolled 5,988 patients with New York Heart Association (NYHA) class II-IV HF and left-ventricular ejection fraction >40% (median EF ≈54%), of whom about 49% had T2DM. Over a median follow-up of 26.2 months (≈2.2 years), the primary composite of CV death or HF hospitalization occurred in 13.8% of the empagliflozin group versus 17.1% of placebo, yielding an HR of 0.79 (95% CI 0.69-0.90; P<0.001). This benefit was driven mainly by a 27% reduction in HF hospitalizations (HR 0.73) [52]. Thus, empagliflozin reduced major HF outcomes in this largely untreated HFpEF/mid-range EF population, supporting a disease-modifying effect across the EF spectrum.

Finally, DECLARE-TIMI 58, the largest SGLT2 inhibitor CV outcomes trial, enrolled 17,160 T2DM patients (40% established CVD, 60% multiple risk factors) receiving dapagliflozin 10 mg or placebo for 4.2 years. While MACE was neutral (HR 0.93, 95% CI 0.84-1.03), dapagliflozin significantly reduced CV death/HF hospitalization (HR 0.83, 95% CI 0.73-0.95), driven by 27% fewer HF admissions (HR 0.73, 95% CI 0.61-0.88), plus 24% lower renal events (HR 0.76) [53].

Clinical evidence of SGLT2 inhibitors’ renal benefits

Across CKD populations, SGLT2 inhibitors consistently demonstrate strong kidney-protective and CV benefits (Table 2). In broad CKD populations, the EMPA-KIDNEY trial evaluated empagliflozin 10 mg daily in patients with CKD, with or without T2DM, on standard background therapy, with a median follow‑up of about 2.0 years.

**Table 2:** Key clinical trials of SGLT2 inhibitors on renal outcomes across the glycemic spectrum SGLT2: sodium-glucose cotransporter 2; CKD: chronic kidney disease; CV: cardiovascular; eGFR: estimated glomerular filtration rate; ESKD: end-stage kidney disease; FU: follow-up; HFH: heart failure hospitalization; HR: hazard ratio; T2DM: type 2 diabetes mellitus

Trial	n	T2DM (%)	Median FU (years)	Key findings	Main results
EMPA-KIDNEY [54]	6,609	46	2.0	Reduced kidney disease progression and CV death in the broad CKD population. Slowed eGFR decline	Reduced kidney/CV progression in CKD±T2DM Primary composite: HR 0.72 (0.64-0.82)
CREDENCE [55]	4,401	100	2.62	Reduced ESKD and CV events in T2DM with CKD	Renal/CV composite: HR 0.70 (0.59-0.82) HFH: HR 0.61 (0.47-0.80)
DAPA-CKD [56]	4,304	67	2.4	Slowed eGFR decline and reduced renal risk	Reduced kidney/CV events in CKD±T2DM Primary composite: HR 0.61 (0.51-0.72) Renal composite: HR 0.50 (0.41-0.61) All-cause death: HR 0.69 (0.53-0.88)

Empagliflozin significantly reduced the risk of the composite primary outcome of kidney disease progression (defined as end-stage kidney disease (ESKD), a sustained ≥40% decline in eGFR, or renal death) or CV death (HR approximately 0.72; 95% CI roughly 0.64-0.82). It also slowed chronic eGFR decline and reduced hospitalizations for any cause, with broadly consistent benefits across key subgroups, including those without diabetes [54]. The CREDENCE trial evaluated canagliflozin in patients with T2DM and albuminuric CKD receiving background RAAS blockade, with a median follow-up of 2.62 years.

Canagliflozin significantly reduced the risk of the composite of ESKD, doubling of serum creatinine, or death from renal causes (HR 0.70; 95% CI 0.59-0.82). Renal-specific outcomes were also reduced, including the composite of ESKD, doubling of creatinine, or renal death (HR 0.66; 95% CI 0.53-0.81) and ESKD alone (HR 0.68; 95% CI 0.54-0.86) [55]. The DAPA-CKD trial evaluated dapagliflozin 10 mg daily in patients with CKD, both with and without T2DM, already receiving stable renin-angiotensin system blockade, with a median follow‑up of about 2.4 years.

Dapagliflozin significantly reduced the risk of the composite primary outcome of ≥50% sustained decline in eGFR, ESKD, or death from renal or CV causes (HR approximately 0.61; 95% CI roughly 0.51-0.72). Renal-specific outcomes were also reduced, including the composite of sustained ≥50% eGFR decline, ESKD, or renal death, and the drug lowered all-cause mortality and hospitalization for HF in this CKD population [56].

Safety profile of SGLT2 inhibitors

**Figure 1:**
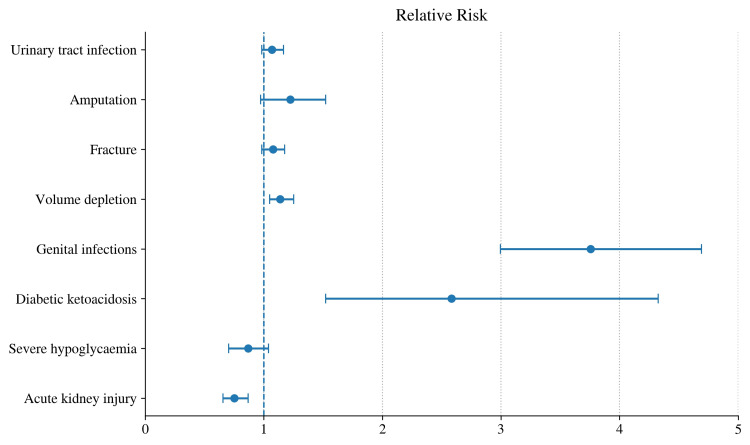
Forest plot summarizing the safety of four SGLT2 inhibitors (dapagliflozin, empagliflozin, ertugliflozin, and canagliflozin) across patients with type 2 diabetes, chronic heart failure, and chronic kidney disease Meta-analysis (nine RCTs, n=59,692): Fracture 1.07 (0.99-1.16); DKA 2.57 (1.53-4.31); Amputation 1.21 (0.97-1.51); UTI 1.07 (0.99-1.15); Genital infection 3.75 (3.00-4.67); AKI 0.75 (0.66-0.85); Severe hypoglycemia 0.86 (0.71-1.03); Volume depletion 1.14 (1.05-1.24) Source: Data were extracted from a single study [57] and are presented for illustrative purposes. SGLT2: sodium-glucose cotransporter-2; RCTs: randomized controlled trials; DKA: diabetic ketoacidosis; UTI: urinary tract infection; AKI: acute kidney injury

SGLT2 inhibitors, including dapagliflozin, empagliflozin, ertugliflozin, and canagliflozin, demonstrate a generally favorable safety profile (Figure 1) [57]. Supported by other meta-analyses, SGLT2 inhibitors are generally well tolerated across patients with T2DM, chronic HF, and CKD [58,59]. Several predictable possible side effects have been documented and should be considered when prescribing this class of medication. These adverse events are mechanism-based, resulting from the promotion of urinary glucose excretion. Genital mycotic infections are the most frequently reported complication, while urinary tract infections are modestly increased, although serious events remain uncommon [48].

An increased risk of lower-limb amputation was initially observed in the CANVAS Program. In contrast, the CREDENCE trial did not demonstrate a significant difference in amputation risk, with similar event rates between the canagliflozin and placebo groups [55]. Likewise, no consistent amputation signal was observed in EMPA-REG OUTCOME or DECLARE-TIMI 58 [44,53]. Although the canagliflozin signal remains noted in safety discussions, its relevance to the broader SGLT2 inhibitor class remains uncertain.

Future perspectives

SGLT2 inhibitors have emerged as a transformative therapy for patients with T2DM, HF, and CKD, demonstrating consistent cardiorenal protection across diverse populations [16,5]. These benefits are mediated via cellular stress and inflammation by modulating inflammatory signalling pathways and enhancing cellular function and resilience [60,61]. However, important limitations remain. Patients with eGFR <30 mL/min/1.73 m^2^ were underrepresented in most trials, and those included were often analyzed only as small subgroups, which may reduce the certainty of conclusions. Importantly, patients with stage 5 CKD or those on dialysis were not enrolled in any major SGLT2 inhibitor trials, leaving a gap in evidence regarding safety and efficacy in this population [11,12,55,62,63]. Consequently, while current data support the use of SGLT2 inhibitors in low eGFR patients [64], further studies are needed to determine their effects in stage 5 CKD and dialysis-dependent individuals.

Although empagliflozin modestly lowered serum uric acid in EMPA-KIDNEY, this did not translate into a significant reduction in gout events, suggesting that the clinical relevance of uric acid reduction by SGLT2 inhibitors requires further study [62]. In this relatively low-risk population, early initiation of dapagliflozin following MI did not result in significant short-term reductions in MACE [49]. These findings suggest that the cardioprotective effects of SGLT2 inhibitors may be less pronounced in the acute phase among patients without overt comorbidities. A longer follow-up may be necessary to determine whether delayed benefits emerge in this subgroup.

Further applications and perspectives in various fields and medical issues are still being researched. Thorough research of this medication class at the experimental and clinical levels may help to uncover potential positive benefits of gliflozins in patients with hepatological disorders (such as non-alcoholic fatty liver disease), valvular heart disease, atrial fibrillation, and acute HF. Cardiogenic shock, hypertrophic cardiomyopathy, glomerulonephritis, and severe renal disease are among the other disorders that are now under investigation [24,65].

Limitations

We acknowledge some limitations to this review. First, although we performed a structured search of PubMed and Google Scholar and synthesized English-language evidence published between January 2019 and December 2025, the approach was a narrative review rather than a formal systematic review with meta-analysis; therefore, selection and publication biases cannot be excluded, and the certainty of inference may vary across outcomes and populations. Second, substantial heterogeneity across included studies (baseline CV risk, HF phenotype, CKD stage, endpoints, follow-up duration, and background therapies) limits direct cross-trial comparisons. Third, several mechanistic conclusions rely heavily on preclinical and short-term physiological evidence, so causal links to long-term clinical outcomes remain partly inferential. Finally, important evidence gaps persist - particularly for patients with eGFR <30 mL/min/1.73 m^2^, stage 5 CKD, and dialysis-dependent populations - and early post-MI use in relatively lower-risk cohorts has not consistently reduced short-term hard endpoints, underscoring the need for dedicated trials and longer follow-up.

## Conclusions

Growing evidence shows that SGLT2 inhibitors act as disease-modifying therapies with benefits that go far beyond their original role. SGLT2 inhibitors demonstrate a favorable balance of efficacy and safety across diverse patient populations. The literature has provided insights into the benefits of SGLT2 inhibitors on cardiorenal protection, supporting their growing role in improving long-term outcomes for a wide range of patients.
